# The clinically applied PARP inhibitor talazoparib ameliorates imiquimod-induced psoriasis in mice without reducing skin inflammation

**DOI:** 10.3389/fphar.2025.1519066

**Published:** 2025-02-19

**Authors:** Petra Molnár, Máté Ágoston Demény, Beáta Várkonyi, Zsuzsanna Polgár, Ágnes Pór, Ilona Kovács, Andrea Szegedi, Attila Gábor Szöllősi, Magdolna Szántó

**Affiliations:** ^1^ Department of Immunology, Faculty of Medicine, University of Debrecen, Debrecen, Hungary; ^2^ Department of Medical Chemistry, Faculty of Medicine, University of Debrecen, Debrecen, Hungary; ^3^ National Academy of Scientist Education, University of Debrecen, Debrecen, Hungary; ^4^ Department of Pathology, Gyula Kenézy Campus, Clinical Centre, University of Debrecen, Debrecen, Hungary; ^5^ Department of Dermatology, Faculty of Medicine, University of Debrecen, Debrecen, Hungary; ^6^ HUN-REN-UD Allergology Research Group, University of Debrecen, Debrecen, Hungary

**Keywords:** PARP inhibitor, psoriasis, terminal differentiation, apoptosis, mitochondria, drug repurposing

## Abstract

**Background:**

Considering the role PARPs play in inflammation, we assessed the effect of PARP inhibition in an inflammatory skin condition, psoriasis, to explore novel avenues for the potential repurposing of PARP inhibitors that are currently used in tumour therapy.

**Methods:**

The imiquimod (IMQ)-induced model of psoriasis was applied in BALB/c mice. Mice received daily intraperitoneal injection of either one of four PARP inhibitors or their vehicle prior to treatment of the shaved back skin of mice with IMQ-containing cream or control cream for four days. The appearance of the skin of mice was scored daily according to the extent of erythema, induration and scaling. The most effective PARP inhibitor was selected for detailed studies on mouse skin and in a human keratinocyte cell line.

**Results:**

Of the PARP inhibitors, talazoparib and rucaparib improved the imiquimod-induced symptoms on mouse skin. Application of talazoparib in the psoriasis model resulted in maintained terminal differentiation and reduced proliferation of epidermal keratinocytes. Conversely, talazoparib also enhanced the production of pro-inflammatory chemokines in the skin of mice. These effects of talazoparib was associated with increased mitochondrial production of reactive oxygen species and a consequent activation of pro-apoptotic and pro-inflammatory pathways in keratinocytes.

**Conclusion:**

PARP inhibition by talazoparib promotes terminal differentiation of epidermal keratinocytes that may be beneficial in psoriasis. Despite the fact that talazoparib exerted a pro-inflammatory effect in the skin, which is not unprecedented in anti-psoriatic therapy, these findings may advance the conduction of pre-clinical and clinical trials with PARP inhibitors in psoriasis management.

## Introduction

PARP activation was first described in relation to DNA damage response, during which PARP1 cleaves NAD^+^ to yield ADP-ribose residues that it transfers to target proteins, building up poly (ADP-ribose) (PAR) chains (Chambon et al., 1963; Durkacz et al., 1980; Miller, 1975). A couple of decades later, a second PARP enzyme, PARP2 was shown to exert similar catalytic activity (Ame et al., 1999; Schreiber et al., 2002). In the early 2000s, it was recognized that tumour cells with defective homologous recombination DNA repair pathways, such as BRCA1/2-deficient cancer cells, are highly sensitive to the inhibition of PARP activity (Bryant et al., 2005; Farmer et al., 2005). That realization gave a significant boost to research and development efforts with PARP inhibitors, and currently olaparib, rucaparib, niraparib and talazoparib have FDA approval as anti-cancer agents. All of these PARP inhibitors display simultaneous inhibition of PARP1 and PARP2 (Knezevic et al., 2016; Thorsell et al., 2017), the two isoforms with highest activity, as PARP1 covers 85%–95%, while PARP2 covers 5%–15% of total cellular PARP activity depending on the model used (Ame et al., 1999; Schreiber et al., 2002).

As a result of intense studies conducted on the PARP field during the past decade, now ample evidence demonstrates that PARP activation and PAR synthesis are involved in a plethora of biological processes beyond DNA repair. These processes involve energy metabolism and mitochondrial biology (Szanto and Bai, 2020), lipid metabolism (Szanto et al., 2021), transcriptional and translational regulation (Jubin et al., 2017), cell death (Fatokun et al., 2014; Hong et al., 2004), differentiation and inflammation (Fehr et al., 2020; Kunze and Hottiger, 2019). In accordance, the clinically used PARP inhibitors displayed beneficial effects in several models of non-oncological, inflammatory pathologies, such as asthma (Ghonim et al., 2015), sepsis (Ahmad et al., 2019), alcoholic hepatitis (Gariani et al., 2017; Mukhopadhyay et al., 2017), acute pancreatitis (Ahmad et al., 2020), burn injury (Ahmad et al., 2018), and acute lung injury (Martins et al., 2022). Hence, it has been postulated that PARP inhibitors could be repurposed for the treatment of non-oncological diseases (Berger et al., 2018).

Psoriasis is one the most common inflammatory skin diseases, to which currently there is no cure. Psoriasis manifests under specific genetic and environmental factors, and it is characterized by the hyperproliferation and aberrant terminal differentiation of epidermal keratinocytes. This results in the formation of thickened, red plaques on various skin areas. (Griffiths et al., 2021). Psoriatic skin lesions contain a robust immune cell infiltrate, and psoriasis is considered primarily an immune-mediated disorder. It is widely accepted that T helper (Th)17 cells and the IL17 cytokine family has a prominent role in activating those signalling circuits in keratinocytes that sustain chronic inflammation in psoriasis (Antal et al., 2022; Lowes et al., 2007).

Psoriasis has several animal models, among them, the most widely utilized in psoriasis-related studies is the imiquimod (IMQ)-induced psoriasiform dermatitis (van der Fits et al., 2009). We previously studied this model in *PARP1* ^*−/−*^ and *PARP2* ^*−/−*^ mice. Surprisingly, we found that the dermatitis upon IMQ treatment was more severe in *PARP1* ^*−/−*^ mice than in wild-type mice (Kiss et al., 2020), while *PARP2* ^*−/−*^ mice displayed a protected phenotype against the IMQ-induced symptoms (Antal et al., 2023). In the current study, we aimed to test the effect of PARP inhibitors, which target PARP1 and PARP2 in equal measure, in the IMQ-induced psoriasis model.

## Materials and methods

### Mice

BALB/c mice were bred at the animal facility of the University of Debrecen, Hungary. No more than six mice were housed in each cage (standard block shape 365 × 207 × 140 mm, surface 530 cm^2^; 1284 L Eurostandard Type II. L from Techniplast, Milan, Italy) with Lignocel Select Fine (J. Rettenmaier und Söhne, Rosenberg, Germany) as bedding. Mice had *ad libitum* access to water (sterilized tap water) and food (chow diet - Safe, Augy, France), and were kept under a 12/12-h dark/light cycle. Only male mice were used in the study at the age of 8–10 weeks. Mice had paper tubes to enrich their environment. Cages were changed once a week, on the same day. The animal facility was overseen by a veterinarian.

### Imiquimod-induced psoriasis model

Mice were housed separately during the experiment. Two days prior to the start of the study, the back of mice was shaved in a 2 × 2 cm area, then mice were weighed and divided into different cohorts. Each day, mice received 200 μL intraperitoneal (i.p.) injection of either vehicle (VEH) (solvent composed of 4% dimethyl-sulfoxide, 5% polyethylene glycol 300 in phosphate-buffered saline (PBS)) or one of the following PARP inhibitors: talazoparib (S7048) solution (2 mg kg^-1^·day^-1^); olaparib (S1060), (20 mg kg^-1^·day^-1^) rucaparib (S4948) (50 mg kg^-1^·day^-1^); niraparib (S2741) (10 mg kg^-1^·day^-1^) (all from Selleck Chemicals, Houston, TX, United States) 1 h prior to topical treatment with either 62.5 mg of Aldara cream (5% imiquimod (IMQ), Meda AB, Solna, Sweden) or a control cream (CTL) for four consecutive days. The severity of the lesions, including induration, erythema and scaling, was scored daily by two experienced researchers (one of them was a dermatologist), each on a scale from 0 to 4, where 0 denotes no symptoms and 4 denotes the most severe symptoms. On day 5, 2 h prior to sacrifice, mice received 5-bromo-2-deoxyuridine (ab142567; Abcam, Cambridge, United Kingdom) (BrDU, 100 mg/kg of body weight i. p.), then mice were sacrificed, and skin biopsies were taken for further analyses. The study was approved by the Veterinary Centre of the University of Debrecen, Hungary (study registration No. 5/2023/DEMÁB).

### Histology

Histology analyses were performed on 4 μm thick sections cut from formalin fixed, paraffin embedded mouse skin samples, similar, as in (Antal et al., 2023). Section were stained with hematoxylin and eosin (H&E). For immunohistochemistry (IHC), blocked tissue sections were incubated with specific primary antibodies (RelA/NFkB p65 [p Ser276]: NB100-82086; Involucrin: 924401, Biolegend (San Diego, Ca, United States); Caspase-3: 9662, Cell Signaling Technology (Danvers, MA, United States)). Protein expression was detected with Mouse and Rabbit Specific HRP/DAB (ABC) Detection IHC kit (ab64264; Abcam, Cambridge, United Kingdom) according to the manufacturer’s protocol. For detection of BrdU, a commercial IHC kit was used (ab125306, Abcam). Negative controls were obtained by omitting the primary antibodies. Slides were digitalized with the PANNORAMIC confocal slide scanner (3DHISTECH Ltd., Budapest, Hungary). Scanned slides were studied by the SlideViewer 2.8 for Windows 11 software of 3DHISTECH Ltd. Epidermal thickness of mouse skin was measured in the SlideViewer 2.8 software.

### Cell culture

Cell culture studies were performed on the HPV-Ker human keratinocyte cell line. Human keratinocytes were immortalized with the HPV-E6 oncogene by researchers of the University of Szeged, Hungary in collaboration with Creative Laboratory Ltd., Szeged, Hungary (Tax et al., 2016), and the thereby generated HPV-Ker cells were kindly provided by Creative Laboratory Ltd. Cells were maintained in Keratinocyte serum-free medium (SFM) supplemented with Bovine Pituitary Extract (BPE) (50 μg/mL), human recombinant Epidermal Growth Factor (rEGF) (5 ng/mL) (17005042, Gibco, Thermo-Fisher Scientific, Waltham, MA, United States), 1% penicillin/streptomycin (P4333, Merck Millipore, Burlington, MA, United States) and 1% L-glutamine solutions (59202C, Merck) in 5% CO_2_ humidified air at 37°C. For differentiation of HPV-Ker keratinocytes, post-confluent cells were kept in antibiotics-free culture media supplemented with 1.7 mM Ca^2+^ for 3 days. Cells were exposed to treatments with TAL (100 nM); IL17A (200 ng/mL; 7955-IL, R&D systems, Minneapolis, MN, United States); TNFα (10 ng/mL; GF023, Merck Millipore). Treatment schemes are highlighted in Figure legends.

### Protein extraction and Western blotting

HPV-Ker cells were washed with PBS solution and were then harvested in RIPA lysis buffer (50 mM Tris, 150 mM NaCl, 1% Triton X-100, 0.5% sodium deoxycholate, 0.1% SDS, 1 mM EDTA, 1 mM Na_3_VO_4_, 1 mM NaF, 1 mM PMSF and protease inhibitor cocktail, all from Merck Millipore) on ice. DNA-protein complexes in cell lysates were disrupted by sonication (Branson Sonifier, Emerson Electric, Pasig, Philippines) for 3 × 20 pulses, 15-s intervals. Protein isolates were separated from debris by centrifugation for 10 min at 10,000 x g, 4°C. Protein lysates were boiled with 5x SDS sample buffer (310 mM Tris-HCl, pH 6.8, 50% glycerol, 10% SDS, 100 mM DTT, 0.01% bromophenol blue, all from Merck Millipore) supplemented with 5% 2-mercaptoethanol for 10 min. Protein extracts (20–30 µg) were separated by SDS-PAGE and transferred onto nitrocellulose membranes. Membranes were blocked with 5% BSA in TBS-Tween or 5% non-fat milk in TBS-Tween for 1 h at room temperature and incubated with primary antibodies overnight at 4°C. Membranes were probed with a peroxidase-conjugated secondary antibody for 1 h at room temperature. Signals were visualized by enhanced chemiluminescence reaction and captured by ChemiDocTM Touch Imaging System (Bio-Rad, Hercules, CA, United States). Bands were quantified by densitometry using Fiji ImageJ software (Schindelin et al., 2012). The following antibodies were used in immunoblots: anti-caspase-3 (9662, Cell Signaling Technology, 1:1000), recombinant anti-cytokeratin 10 (Abcam, ab76318, 1:1000), anti-β-actin-peroxidase (Merck, A3854, 1:20000), anti-mouse IgG, HRP-linked (Merck Millipore, A9044, 1:2000), anti-rabbit IgG, HRP-linked (Cell Signaling Technology, 7074S 1:1000).

### Mouse cytokine array

Mouse skin samples were flash frozen in liquid nitrogen after excision. Before protein extraction, tissues were weighed and rinsed with PBS. Tissues were homogenized in PBS containing protease inhibitor cocktail (S8830, Merck Millipore) using 5 mm stainless steel beads at 30 Hz for 8 min in a TissueLyser II device (Qiagen, Hilden, Germany). Afterwards, Triton X-100 was added to the samples to reach 1% final concentration. The samples were then centrifuged at 10,000 × g for 5 min at 4°C to remove any debris. The supernatant was saved, and protein content was quantified using a Pierce BCA protein assay kit (23225, Thermo Fisher Scientific). 300 μg protein of each samples within a treatment group was pooled and used for the mouse cytokine array according to the protocol provided by the manufacturer (Proteome Profiler Array, No. ARY006, R&D Systems/Bio-Techne). The immunoblot images were captured and visualized using a ChemiDoc™ Touch Imaging System (Bio-Rad Laboratories) and the integrated intensity of each spot in the images was analyzed using CellProfiler for non-cell images analysis software (Broad Institute of MIT and Harvard) (Lamprecht et al., 2007).

### Apoptosis measurement

HPV-Ker cells were exposed to various treatments, subsequently apoptotic cells were detected using eBioscience Annexin V Apoptosis Detection Kit FITC with propidium iodide (PI) (88–8005-72/74, Invitrogen, Waltham, MA, United States) according to the manufacturer’s instructions. Briefly, supernatants of cells were removed and put aside. Cells were washed with PBS, trypsinized and washed with the supernatant media complemented with fetal bovine serum (FBS) into polystyrene tubes, then cells were pelleted by centrifugation (2000 rpm, 5 min, 4 °C). Cells were washed again with PBS, centrifuged, and stained with FITC-conjugated Annexin V and PI (5–5 μL/100 μL binding buffer/2 × 10^5^ cells) for 15 min at room temperature. Samples were then diluted with PBS and subsequently analysed by a NovoCyte Flow Cytometer (Agilent Technologies, San Diego, CA, United States).

### Analysis of mitochondrial content

For microscopy, HPV-Ker cells were seeded on coverslips in 24-well plates, and were exposed to various treatments. Cells were incubated in culture media containing 100 nM MitoTracker Red CMXRos dye (M7512, Thermo Fisher Scientific) for 30 min, washed three times with culture media, then cells were fixed with 4% formaldehyde. After washing three times with PBS, nuclei of cells were counterstained with Hoechst 33342 dye (Thermo Fisher Scientific), subsequently, cells were observed in a TCS SP8 confocal microscope (Leica, Wetzlar, Germany). For flow cytometry, HPV-Ker keratinocytes were seeded in 6-well plates. Cells were trypsinized, washed with fetal bovine serum-containing culture media, centrifuged, and incubated in culture media with 100 nM MitoTracker Red CMXRos dye for flow cytometry (M46752, Thermo Fisher Sceintific) for 30 min at 37°C for 30 min. Afterwards, cells were washed twice with PBS, then analyzed by Novocyte Flow Cytometer (Agilent Technologies).

### Analysis of mitochondrial reactive oxygen species (ROS) production

To detect mitochondrial ROS, HPV-Ker cells were seeded in 6-well plates and were subjected to various treatments. Cells were trypsinized, washed with FBS-containing culture media, centrifuged, and incubated in culture media with 5 μM MitoSOX Red mitochondrial superoxide indicator (M36008, Thermo Fisher Scientific) at 37°C for 30 min. Afterwards, cells were washed twice with HBSS buffer, then analyzed by Novocyte Flow Cytometer (Agilent Technologies).

### RNA isolation and quantitative PCR

Total RNA from cells was isolated using PureLink RNA kit (12183018A; Thermo Fisher Scientific) according to the manufacturer’s instructions. RNA samples were treated with Ambion DNase I (AM2222; Thermo Fisher Scientific), afterwards, samples were reverse transcribed using a High-Capacity cDNA Reverse Transcription Kit (4368813; Thermo Fisher Scientific). The RT-qPCR reactions were performed in a Light-Cycler 480 Detection System (Roche, Bazel, Switzerland) using TaqMan assays: ATF3 (Hs00231069_m1); IL6 (Hs00174131_m1); IL1β (Hs01555410_m1); CXCL8 (Hs00174103_m1). RT-qPCR data was quantified by calculating normalized gene expressions using the ΔΔCt method. GAPDH (Hs02786624_g1) was used as an internal control gene for normalization. Taqman assays were from Thermo Fisher Scientific.

## Statistics

The distribution of data was analysed by Shapiro-Wilk test. If two groups were compared, we used independent t-test (two-tailed), as the Shapiro–Wilk test showed normal distribution. If the distribution was not normal, Mann-Whitney test was used. When we compared more than two groups and if the distribution was normal, we used ANOVA followed by Tukey’s *post hoc* test. In case the data did not show a normal distribution, Kruskal–Wallis test were applied complemented by Dunn’s *post hoc* test. The significance level was set at 0.05. Statistical tests were performed using GraphPad Prism 9.1.2 software (GraphPad Software, Boston, MA, United States).

## Results and discussion

To test if PARP inhibition can affect IMQ-induced psoriasis-like dermatitis in mice, first we performed a pilot study with the four clinically available inhibitors. We included only male mice in this study for several reasons. Multiple studies show that psoriasis changes in women during pregnancy, *postpartum* and with menopause, indicating that psoriasis is influenced by estrogen hormones (Adachi and Honda, 2022; Lin and Huang, 2016). In addition, a recent study provided evidence that estradiol suppresses IMQ-induced psoriasis in mice (Adachi et al., 2022). Since female mice have estrous cycle, it is possible that the hormonal status of female mice could interfere with the development and severity of IMQ-induced symptoms. To note, studies suggest that the effects of PARP inhibitors also depend on the sex of the animals, with male mice being more responsive than females (Mabley et al., 2005; McCullough et al., 2005).

**FIGURE 1:**
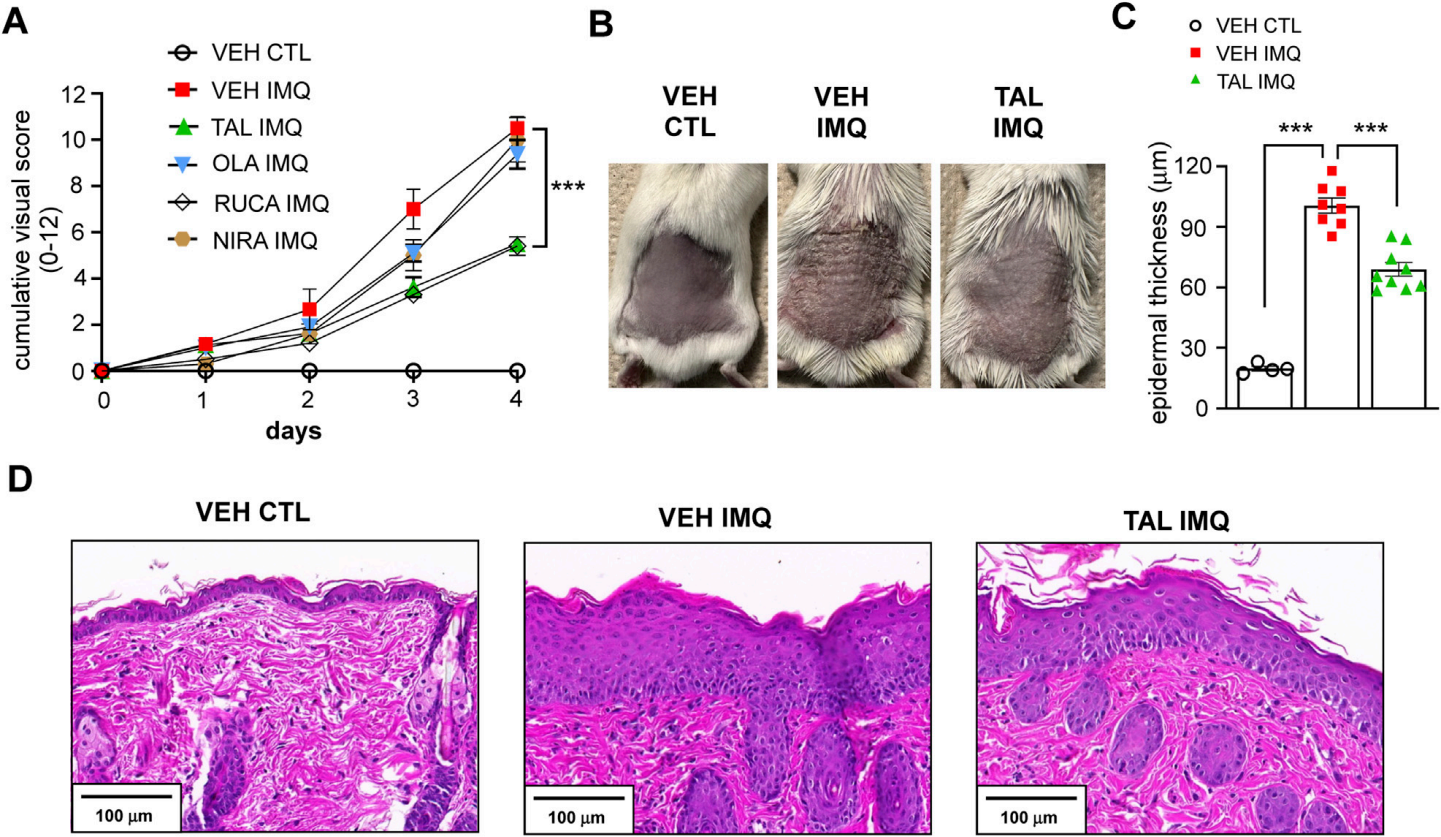
PARP inhibition with TAL alleviates IMQ-induced lesions in mice **(A)** BALB/c mice received i.p. injection of either vehicle (VEH) or one of the listed PARP inhibitors (TAL: talazoparib; OLA: olaparib; RUCA: rucaparib; NIRA: niraparib) prior to treatment with IMQ. The VEH CTL cohort received VEH i. p. injection prior to treatment with a control (CTL) cream. Daily cumulative visual score (sum of scores for erythema, induration and scaling) of IMQ-induced dermatitis is presented as mean ± SEM (VEH CTL: n = 4, VEH IMQ: n = 8, TAL IMQ: n = 9). Significance was calculated using two-way ANOVA followed by Dunnett’s *post hoc* test for multiple comparisons. *** shows statistical difference between VEH IMQ and TAL IMQ groups at p < 0.001. **(B)** Photograph taken on day 5 of the IMQ-induced psoriasis model shows back skin of mice that received TAL i.p. injection (2 mg kg^-1^·day^-1^) and IMQ treatment (TAL IMQ) compared to VEH CTL and VEH IMQ mice. **(C)** Statistical difference between groups was calculated using one-way ANOVA followed by Tukey’s post hoc test, and data is represented as mean ± SEM. ***p < 0.001. **(D)** Hematoxylin and eosin (H&E) staining of skin sections of mice shows the marked thickening of skin on day five of IMQ treatment.

We found in the pilot experiment that, in the applied doses, only rucaparib and talazoparib reduced the extent of induration, erythema and scaling of the skin caused by IMQ treatment, while olaparib and niraparib did not affect the visual appearance of dermatitis compared to the cohort that received only vehicle (VEH) in i. p. injection before treatment of their skin with IMQ (VEH IMQ) (Figure 1A).

It is interesting that not all PARP inhibitors influenced the IMQ-induced psoriasiform lesions. This discordance may have several explanations. For one, we tested the inhibitors in a dosage selected based on oncological studies. However, it has been suggested that the optimal dose of PARP inhibitors in non-oncological conditions may be lower than the doses used in oncological indications (Ahmad et al., 2018), and that the inhibitors may have bell-shaped dose-responses (Teng et al., 2016). Another feasible explanation may be the known difference in the pharmacological and pharmacokinetic profiles of the compounds (Zeng et al., 2024). For example, niraparib and talazoparib have longer half-lives than olaparib and rucaparib, hence only niraparib and talazoparib are used in a once-daily dosing in therapy (Zeng et al., 2024). Talazoparib goes through minimal hepatic metabolism but its dosage should be reduced in patients with impaired renal function, while niraparib requires dose adjustments in patients with hepatic impairment (Zeng et al., 2024). In our experiments we formulated the PARP inhibitors for i. p. injection. Administration via oral gavage may have been closer to the tablet formulation how PARP inhibitors are applied in clinical therapy currently, and may have yielded different results.

Nevertheless, based on our findings and considering that talazoparib is the most potent PARP inhibitor (Curtin and Szabo, 2020), we chose the talazoparib-treated cohort for mechanistic studies. Figure 1B shows the back skin of mice that received either VEH or talazoparib daily i. p. injection prior to topical IMQ treatment (VEH IMQ and TAL IMQ, respectively) as compared to mice that received VEH i. p. and were treated with a control cream (VEH CTL) on day 5 of the experiment. The IMQ-evoked epidermal thickening was less pronounced in the skin of mice that received talazoparib (Figure 1C), as is visible on skin sections after hematoxylin and eosin (H&E) staining (Figure 1D).

**FIGURE 2:**
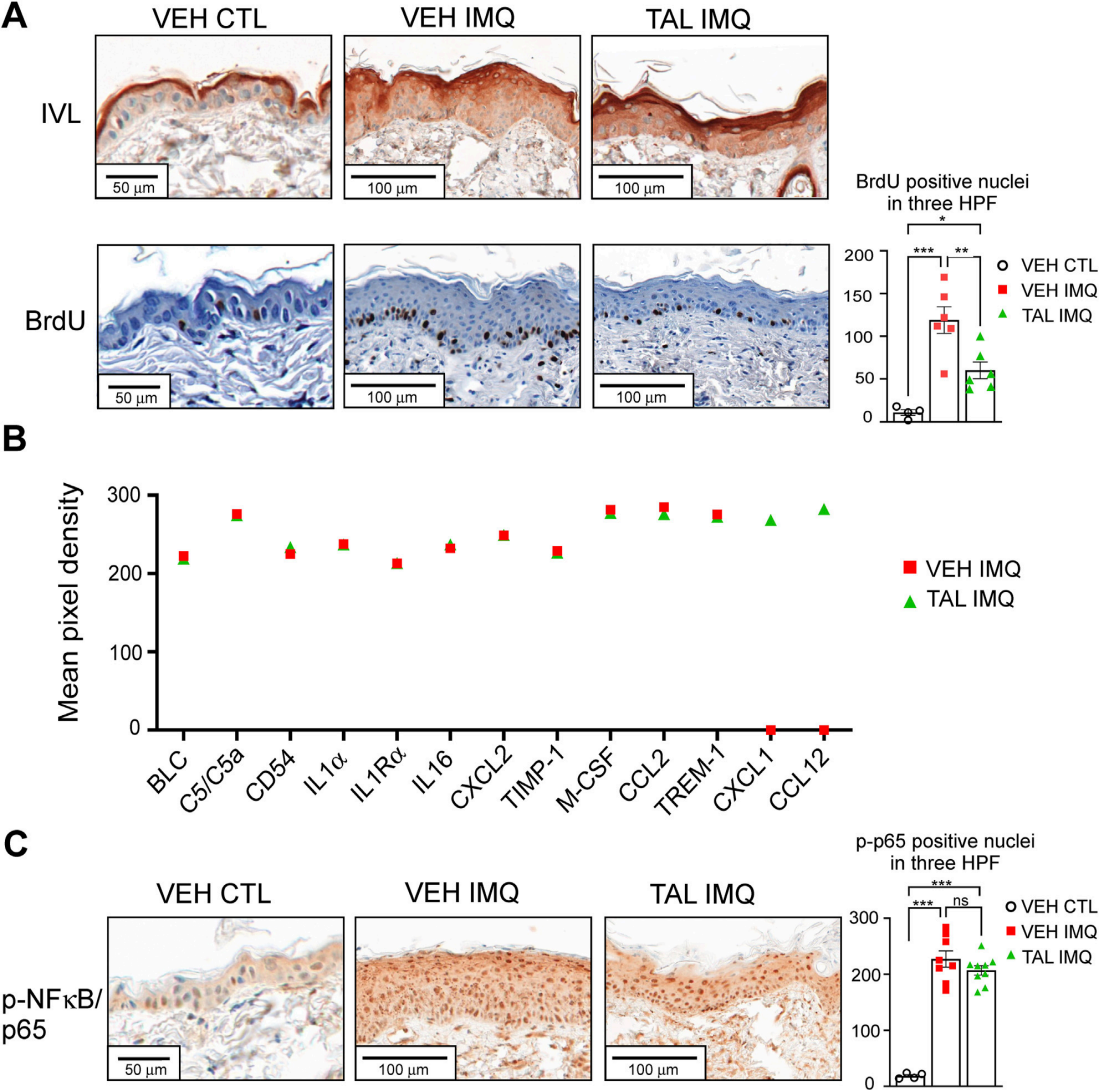
TAL improves terminal differentiation and reduces proliferation, but does not decrease inflammation in psoriasiform lesions **(A)** Involucrin (IVL) IHC was performed on skin sections of CTL or IMQ-treated, VEH or TAL-injected mice. Detection of BrdU incorporation was used to determine the proliferation of epidermal keratinocytes. HPF: high-power field. **(B)** A mouse cytokine array was used to measure inflammatory cytokines and chemokines in whole-skin lysates of mice. Equal amounts of skin protein samples from each of the mice in a given cohort (VEH IMQ or TAL IMQ) were pooled, and the pooled samples were applied on the array membranes. Data represents the mean integrated pixel density of the developed dots from pooled samples corresponding to the listed cytokines on the array membranes. **(C)** NF-κB activity in epidermal keratinocytes of mice was determined in IHC reactions by detecting nuclei positive for phosphorylated p65 subunit of NF-κB. On panels A and C data are presented as mean ± SEM of n = 4-9 mice per cohorts. Statistical significance between cohorts was determined by one-way ANOVA followed by Tukey’s *post hoc* test for multiple comparisons. *p < 0.05, **p < 0.01, ***p < 0.001, ns: not significant.

Immunohistochemical analyses of skin samples revealed premature and smeared expression of the terminal differentiation marker involucrin (IVL) in those mice that received only VEH in i. p. injection before IMQ treatment (VEH IMQ) (Figure 2A). In talazoparib-administered mice (TAL IMQ), although the staining zone broadened, involucrin expression displayed a sharp appearance in the granular layer, more closely resembling normal skin (VEH CTL) (Figure 2A). In addition, analysis of nuclear BrdU incorporation showed fewer proliferating basal keratinocytes in mice that received talazoparib prior to IMQ application (Figure 2A). Next, we performed cytokine array from whole skin lysates. Since visually, the severity of lesions seemed reduced in the talazoparib-treated cohort, it was surprising to find that expressions of inflammatory markers did not change in the skin of these mice compared to the IMQ reference cohort (VEH IMQ). Moreover, inflammation-related chemokines CXCL1 and CCL12 could only have been detected in the samples of talazoparib-treated mice (TAL IMQ), and not in the skin of VEH IMQ mice, suggesting a slight pro-inflammatory effect of talazoparib in the skin (Figure 2B). In accordance, talazoparib did not lessen IMQ-evoked NF-κB activation as determined by detection of phosphorylated p65 positive nuclei in skin sections (Figure 2C).

Given that PARP1 has much higher average activity than PARP2 (85%–90% compared to 5%–15% of total cellular PARP activity), it may be interesting how the phenotype resulted from talazoparib application in the IMQ model channels more aspects of the *PARP2* ^*−/−*^ phenotype than the *PARP1* ^*−/−*^ phenotype in the same model. Namely, the skin of *PARP2* ^*−/−*^ mice similarly displayed maintained terminal differentiation, reduced proliferation and overall alleviated symptoms, but also reduced inflammation after IMQ application compared to wild-type mice (Antal et al., 2023). Conversely, *PARP1* ^*−/−*^ mice displayed more severe psoriasis-like dermatitis and enhanced inflammatory marker expression than the wild-type mice in the IMQ model (Kiss et al., 2020). In this regard, it has to be noted that not all functions of PARPs depend on their catalytic activity, some of their functions rely only on their physical presence and interactions with other proteins (Thomas and Tulin, 2013). The latter functions can only be observed in knock-out model systems and are out of the scope of PARP inhibitors. Characterization of enzymatic activity dependent and independent roles of PARP1 and PARP2 in psoriasis should be elucidated in future studies in order to better understand what signalling mechanisms could be targeted by PARP inhibitors that are relevant in psoriasis.

**FIGURE 3:**
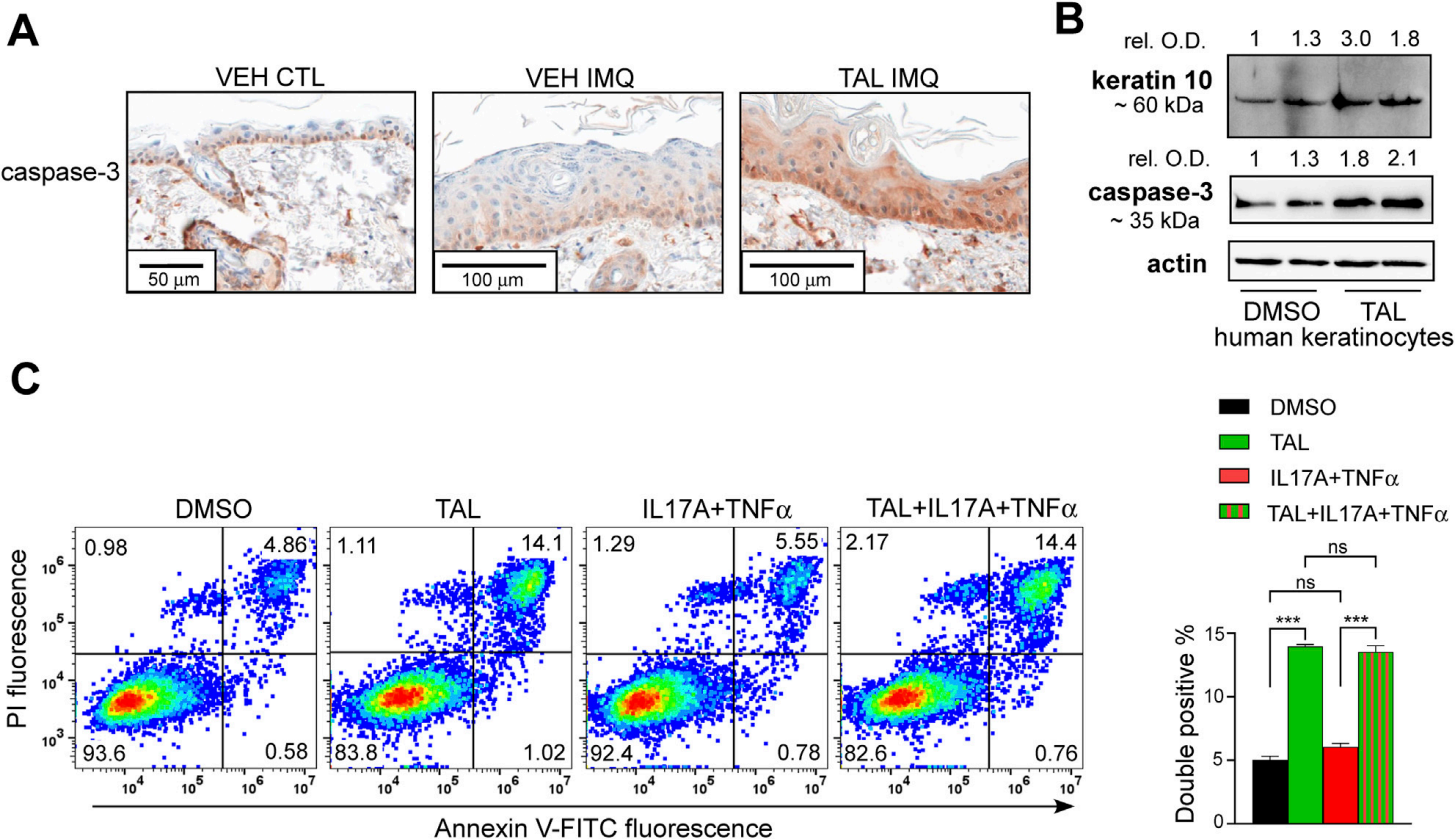
Better terminal differentiation of TAL-treated keratinocytes is associated with higher apoptotic rate **(A)** IHC for apoptotic effector caspase-3 is shown in skin sections of mice that received either VEH or TAL as i.p. injection prior to treatment with either IMQ or CTL cream. **(B)** HPV-Ker human keratinocytes were differentiated in the presence of either dimethyl-sulphoxide (DMSO) or 100 nM TAL for 72 h, and subsequently, keratin 10 and caspase-3 expression was determined by immunoblotting. Optical density values were measured in Fiji ImageJ software, and normalized first to each sample’s internal control (actin), then to the normalized value of the control group. **(C)** Dot plots of flow cytometry double staining by Annexin V-FITC and propidium iodide (PI) and percentages of double-positive cells (right) in differentiated HPV-Ker cells after various treatments. TAL was applied in 100 nM for 72 h. A combination of 200 ng/mL IL17A and 10 ng/mL TNFα was used for stimulation for 24 h. Blot and dot plot images are representatives of three independent experiments, comparisons between groups was made by one-way ANOVA followed by Tukey’s post hoc test, and data are presented as mean ± SEM. ***p < 0.001; ns: not significant.

The phenotype observed upon talazoparib treatment can be explained by its pro-apoptotic effect (Das et al., 2023; Lin et al., 2023), as it has been reported that apoptosis has a crucial role in balancing proliferation and terminal differentiation of epidermal keratinocytes (Raj et al., 2006), and that psoriasis features dysfunctional keratinocyte apoptosis (Kastelan et al., 2009). As the apoptotic effector caspase-3 was shown to be activated during keratinocyte apoptosis and terminal differentiation (Allombert-Blaise et al., 2003; McGill et al., 2005; Raj et al., 2006), we studied caspase-3 expression in the skin of mice. In VEH CTL mice we detected caspase-3 positivity in the basal and lower spinous layers, which was similar in IMQ-treated mice receiving VEH i. p. injection (Figure 3A). In the TAL IMQ cohort, caspase-3 showed strong and even expression in epidermal layers (Figure 3A), indicating enhanced apoptosis in the skin upon talazoparib administration.

**FIGURE 4:**
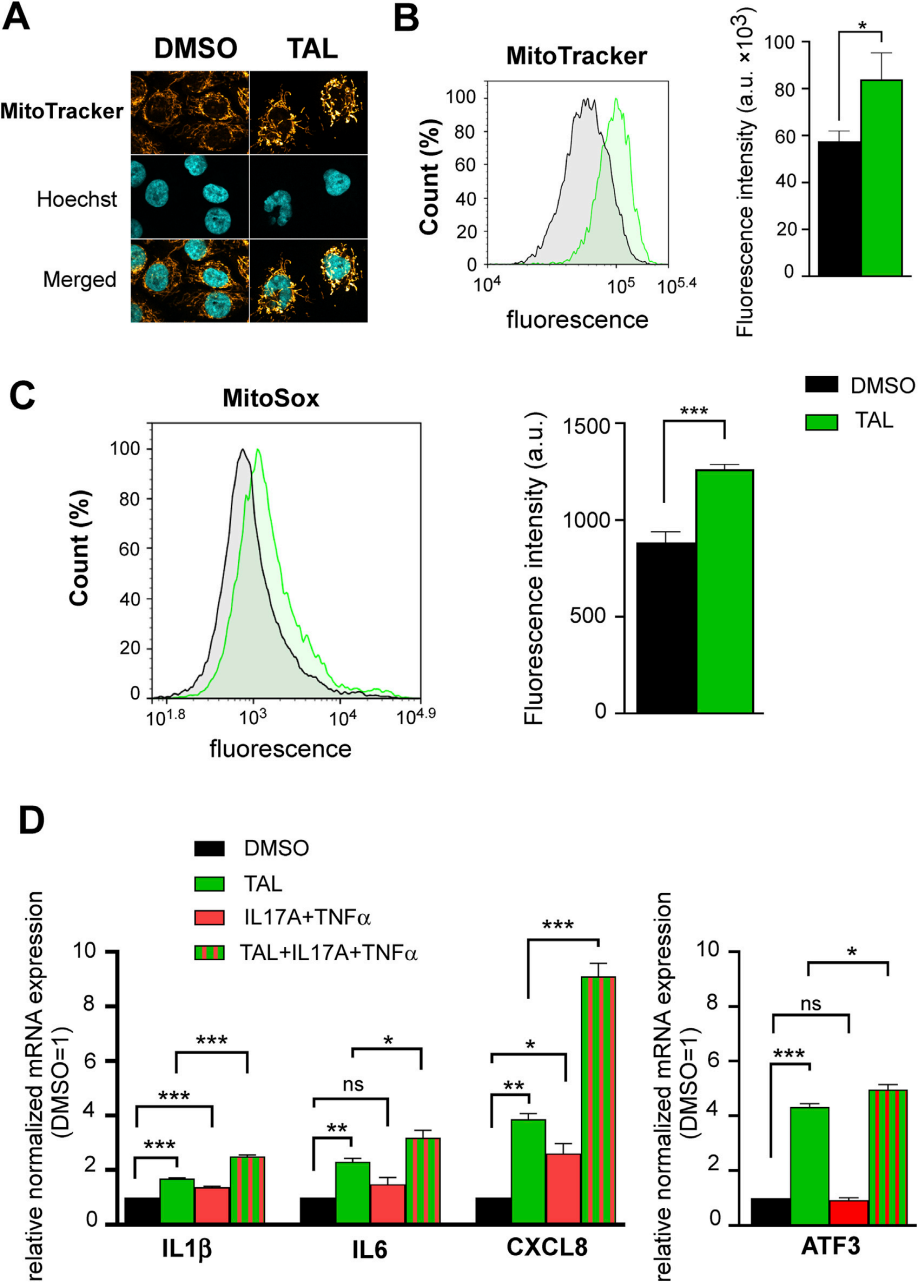
PARP inhibition with TAL promotes mitochondrial function and induces a pro-inflammatory phenotype **(A)** Confocal microscopy images show MitoTracker staining of HPV-Ker keratinocytes treated with either DMSO or 100 nM TAL for 72 h **(B–C)** Histograms and bar charts show MitoTracker and MitoSox fluorescence intensity measured by flow cytometry in HPV-Ker cells subjected to either DMSO or 100 nM TAL for 72 h , statistical comparisons between groups were made by independent t-tests. **(D)** HPV-Ker cells were cultured in the presence or absence of DMSO or 100 nM TAL for 72 h, and in the presence or absence of 200 ng/mL IL17A and 10 ng/mL TNFα for 24 h. Then, total RNA was isolated and subjected to quantitative real-time PCR. Significance was calculated using one-way ANOVA followed by Tukey’s post hoc test. Data are presented as mean ± SEM of three replicates of three independent measurements. For normalization, GAPDH expression was used in the samples. In each experiment, normalized gene expressions in the DMSO (vehicle)-treated samples were set at one, and fold changes relative to DMSO-treated samples are expressed in each treatment conditions. *p < 0.05, **p < 0.01, ***p < 0.001, ns: not significant.

Next, we aimed to study the role of talazoparib in human keratinocyte differentiation. Differentiation of HPV-Ker human keratinocyte cultures in talazoparib-containing media resulted in higher expression of terminal differentiation marker keratin 10, which was accompanied by increased caspase-3 expression (Figure 3B). In addition, Annexin V/propidium iodide dual staining of differentiated keratinocytes revealed an increment in late apoptotic population when the cells were under talazoparib stimulation either alone, or in combination with psoriasis-mimicking cytokines IL17A and TNFα (Figure 3C). These data indicate that talazoparib improves keratinocyte differentiation by activating caspase-mediated programmed cell death. Then we further studied the possible underlying mechanisms of these findings. We have previously shown that PARP1 and PARP2 regulate mitochondrial biogenesis (Bai et al., 2011a; Bai et al., 2011b). Since higher mitochondrial content correlates with greater tendency of cells to undergo caspase-mediated apoptosis (Marquez-Jurado et al., 2018), we studied mitochondrial parameters in keratinocytes upon PARP inhibition with talazoparib. We detected higher MitoTracker (Figures 4A, B) and MitoSox (Figure 4C) fluorescence in talazoparib-treated HPV-Ker keratinocytes, indicative of increased mitochondrial mass and higher mitochondrial production of reactive oxygen species (ROS). Also, we found that talazoparib treatment caused a significant upregulation in pro-inflammatory cytokine mRNA expressions in human keratinocytes, which was even more pronounced when talazoparib was applied together with the combination of IL17A and TNFα (Figure 4D). Although this pro-inflammatory effect of PARP inhibition is somewhat surprising, it is not without precedent. We have previously reported that a combined treatment of HPV-Ker cells with olaparib and IMQ caused higher increase in IL6 mRNA expression than IMQ alone (Kiss et al., 2020). In a different study, olaparib enhanced UVB-induced TNFα and IL8 mRNA expression in human keratinocytes (Chiu et al., 2021). Moreover, olaparib treatment increased ROS production in human tumour macrophages, which essentially promoted pro-inflammatory and anti-tumour function of macrophages (Wang et al., 2022). The exact molecular mechanisms through which talazoparib increased both terminal differentiation and inflammation in keratinocytes is not yet clarified. In this regard, it may be important that we found a marked elevation in the expression of the stress-responsive transcription factor activating transcription factor 3 (ATF3) in talazoparib-treated keratinocytes (Figure 4E). ATF3, depending on context, can either positively or negatively regulate various signalling pathways involved in apoptosis and inflammation (Liu et al., 2024). Mitochondrial ROS-induced ATF3 has been associated with the induction of caspase-3-mediated apoptosis and pro-inflammatory response (Aung et al., 2016; Mashima et al., 2001; Nyunt et al., 2019). We assume that higher ROS production in talazoparib-treated keratinocytes caused the upregulation of ATF3, and consequently, the activation of caspase-mediated apoptosis and induction of inflammatory gene expression. Ultimately, higher apoptotic rate led to better terminal differentiation of keratinocytes, which, at least partly, could explain the amelioration of IMQ-induced symptoms in mice. The pro-inflammatory response observed upon talazoparib treatment calls into question the overall beneficial effect of talazoparib in the IMQ-induced psoriasis model. However, the afore-presented mode of action of talazoparib is very similar to what was described in the case of the commonly used anti-psoriatic topical drug dithranol (anthralin). That is, dithranol was shown to act by inducing mitochondria-driven apoptotic pathways (George et al., 2013; Holstein et al., 2017; McGill et al., 2005), while also producing skin inflammation, due to the generation of ROS (Lange et al., 1998; Peus et al., 2004).

Taken together, this study raises the notion that PARP inhibitors may have an anti-psoriatic effect, but several questions remain unanswered. First, from a translational point of view, it may be a limitation to our study that we used a co-treatment approach with PARP inhibitors and IMQ, through which talazoparib inhibited the full development of psoriatic symptoms, but that does not answer the question if PARP inhibitors could be effective in alleviating an already developed psoriasis, which is when treatment of the disease is typically initiated. We used this approach mainly because one of our aims with this study was to compare the application of PARP inhibitors in mice with the application of *PARP1* ^*−/−*^ and *PARP2* ^*−/−*^ mice that we used in our previous studies in the IMQ model. This approach may more closely mimic the potential preventive effects of PARP inhibitors on psoriasis flares and on the development of more serious symptoms.

Second, it has to be stressed that in this work only keratinocyte-related effects were studied, despite the fact that talazoparib was administered in i. p. injection in mice. Psoriasis is considered primarily an immune-mediated disease. Since PARP1 and PARP2 regulate various aspects of innate and adaptive immune responses (Rosado et al., 2013; Szanto et al., 2024), the skin phenotype we have seen in the psoriasis model in mice upon talazoparib treatment probably involves both immune-modulatory and the hereby presented keratinocyte-specific effects of talazoparib. How PARP inhibition affects immune responses in psoriatic conditions has to be determined in further studies in order to fully evaluate the potential of PARP inhibitor repurposing in psoriasis.

## Data Availability

The original contributions presented in the study are included in the article. Further inquiries can be directed to the corresponding author.
